# Bis­(4-fluoro­benzoato)-κ^2^ *O*,*O*′;κ*O*-(4-fluoro­benzoic acid-κ*O*)bis­(nico­tinamide-κ*N* ^1^)copper(II)

**DOI:** 10.1107/S1600536811020897

**Published:** 2011-06-11

**Authors:** Hacali Necefoğlu, Füreya Elif Özbek, Vijdan Öztürk, Barış Tercan, Tuncer Hökelek

**Affiliations:** a Department of Chemistry, Kafkas University, 36100 Kars, Turkey; b Department of Physics, Karabük University, 78050 Karabük, Turkey; c Department of Physics, Hacettepe University, 06800 Beytepe, Ankara, Turkey

## Abstract

In the title Cu^II^ complex, [Cu(C_7_H_4_FO_2_)_2_(C_7_H_5_FO_2_)(C_6_H_6_N_2_O)_2_], the Cu^II^ cation is coordinated by two N atoms of two nicotinamide (NA) ligands, and by four O atoms from two 4-fluoro­benzoate (PFB) anions and one 4-fluoro­benzoic acid (PFBA) mol­ecule, in a distorted octa­hedral geometry. In the mol­ecule, two Cu—O bond lengths are significantly longer than the other two. The dihedral angles between the carboxyl­ate groups and the adjacent benzene rings are 11.08 (14), 7.62 (13) and 5.73 (11)°, while the benzene rings are oriented at dihedral angles of 15.62 (6), 33.71 (8) and 26.60 (8)°. In the crystal structure, extensive N—H⋯O, C—H⋯F and C—H⋯O hydrogen bonds link the mol­ecules into a three-dimensional network. π–π contacts between the benzene rings [centroid-to-centroid distances = 3.5517 (15), 3.8456 (14) and 3.9265 (13) Å] further stabilize the crystal structure.

## Related literature

For background literature on niacin, see: Krishnamachari (1974 ▶). For information on the nicotinic acid derivative *N*,*N*-diethyl­nicotinamide, see: Bigoli *et al.* (1972 ▶). For related structures, see: Greenaway *et al.* (1984 ▶); Hökelek *et al.* (2010*a* ▶,*b* ▶,*c* ▶,*d* ▶,*e* ▶); Hökelek *et al.* (2009*a* ▶,*b* ▶).
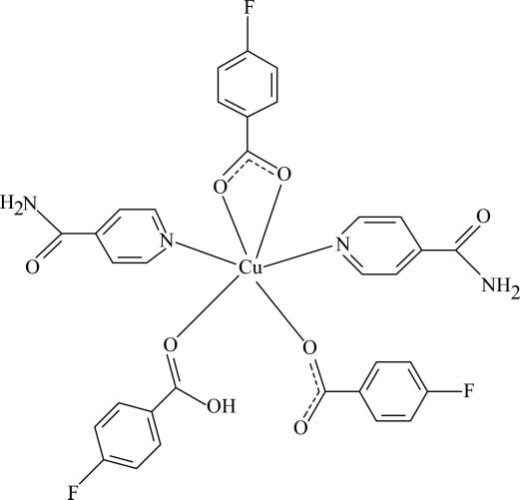


## Experimental

### 

#### Crystal data

[Cu(C_7_H_4_FO_2_)_2_(C_7_H_5_FO_2_)(C_6_H_6_N_2_O)_2_]
*M* *_r_* = 726.12
Triclinic, 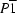

*a* = 10.3370 (2) Å
*b* = 11.6707 (3) Å
*c* = 14.1121 (4) Å
α = 110.824 (4)°
β = 101.333 (3)°
γ = 95.761 (2)°
*V* = 1533.09 (8) Å^3^
*Z* = 2
Mo *K*α radiation
μ = 0.79 mm^−1^
*T* = 100 K
0.48 × 0.32 × 0.24 mm

#### Data collection

Bruker APEXII Kappa CCD area-detector diffractometer
Absorption correction: multi-scan (*SADABS*; Bruker, 2005 ▶) *T*_min_ = 0.745, *T*_max_ = 0.827
27657 measured reflections
7686 independent reflections
6584 reflections with *I* > 2σ(*I*)
*R*_int_ = 0.031

#### Refinement

*R*[*F* ^2^ > 2σ(*F* ^2^)] = 0.036
*wR*(*F* ^2^) = 0.087
*S* = 1.08
7686 reflections
461 parameters
1 restraint
H atoms treated by a mixture of independent and constrained refinement
Δρ_max_ = 0.59 e Å^−3^
Δρ_min_ = −0.53 e Å^−3^

### 

Data collection: *APEX2* (Bruker, 2007 ▶); cell refinement: *SAINT* (Bruker, 2007 ▶); data reduction: *SAINT*; program(s) used to solve structure: *SHELXS97* (Sheldrick, 2008 ▶); program(s) used to refine structure: *SHELXL97* (Sheldrick, 2008 ▶); molecular graphics: *ORTEP-3 for Windows* (Farrugia, 1997 ▶) and Mercury (Macrae *et al.*, 2008 ▶); software used to prepare material for publication: *WinGX* (Farrugia, 1999 ▶) and *PLATON* (Spek, 2009 ▶).

## Supplementary Material

Crystal structure: contains datablock(s) I, global. DOI: 10.1107/S1600536811020897/xu5224sup1.cif

Structure factors: contains datablock(s) I. DOI: 10.1107/S1600536811020897/xu5224Isup2.hkl

Additional supplementary materials:  crystallographic information; 3D view; checkCIF report

## Figures and Tables

**Table 1:** Selected bond lengths (Å)

Cu1—O1	2.0661 (13)
Cu1—O2	2.4581 (14)
Cu1—O3	2.2397 (14)
Cu1—O5	1.9701 (14)
Cu1—N1	2.0024 (15)
Cu1—N3	2.0084 (15)

**Table 2:** Hydrogen-bond geometry (Å, °)

*D*—H⋯*A*	*D*—H	H⋯*A*	*D*⋯*A*	*D*—H⋯*A*
N2—H2*A*⋯O7^i^	0.83 (3)	2.17 (4)	2.990 (3)	175 (3)
N2—H2*B*⋯O2^ii^	0.90 (3)	2.07 (3)	2.943 (2)	163 (3)
N4—H4*A*⋯O1^iii^	0.79 (3)	2.18 (3)	2.953 (2)	165 (3)
N4—H4*B*⋯O8^iv^	0.80 (3)	2.08 (3)	2.880 (3)	174 (2)
O4—H41⋯O6	0.85 (3)	1.62 (3)	2.457 (2)	169 (3)
C4—H4⋯F2^v^	0.93	2.50	3.248 (3)	137
C23—H23⋯O2^ii^	0.93	2.42	3.333 (2)	167
C25—H25⋯O8^vi^	0.93	2.60	3.275 (2)	130
C31—H31⋯O7^vii^	0.93	2.55	3.251 (3)	132

Symmetry codes: (i) 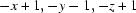; (ii) 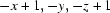; (iii) 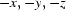; (iv) 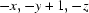; (v) 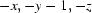; (vi) 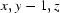; (vii) 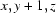.

**Fig. 1.**
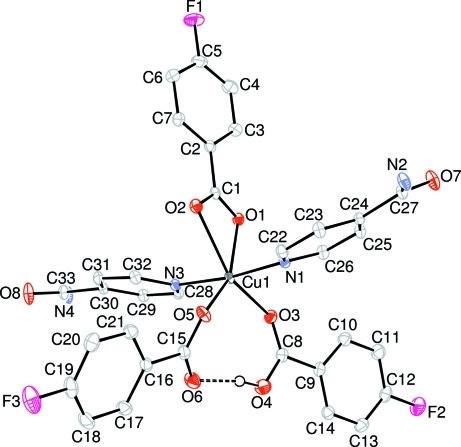
The molecular structure of the title molecule with the atom-numbering scheme. Displacement ellipsoids are drawn at the 50% probability level. The intramolecular O—H···O hydrogen bond is shown as dashed line.

**Fig. 2.**
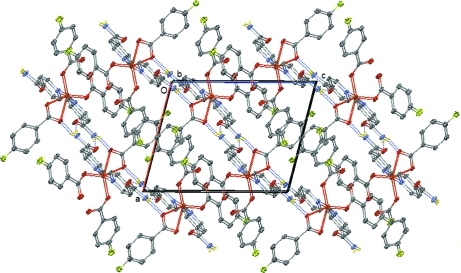
A view of the crystal packing of the title compound. Only the N—H···O hydrogen bonds are shown as dashed lines. [H atoms not involved in hydrogen bonding have been omitted for clarity].

## supplementary crystallographic information

### Comment

As a part of our ongoing investigations of transition metal complexes of nicotinamide (NA), one form of niacin (Krishnamachari, 1974), and/or the nicotinic acid derivative *N*,*N*-diethylnicotinamide (DENA), an important respiratory stimulant (Bigoli *et al.*, 1972), the title compound was synthesized and its crystal structure is reported herein.

The title mononuclear Cu^II^ complex, (I), (Fig. 1), consisting of two nicotinamide (NA), two 4-fluorobenzoate (PFB) and one 4-fluorobenzoic acid (PFBA) ligands. The Cu^II^ center is coordinated by four O atoms from two (PFB) and one (PFBA) ligands, which act in different modes - monodentate, bidentate and monodentate, respectively, and two N atoms of two nicotinamide ligands (Fig. 1). So that, the molecule is six-coordinated. The structures of similar complexes of Cu^II^, Co^II^, Ni^II^ and Zn^II^ ions, [Cu(C_8_H_7_O_2_)_2_(C_6_H_6_N_2_O)_2_].2(H_2_O), (II) (Hökelek *et al.*, 2010*c*), [Co(C_8_H_7_O_3_)_2_(C_6_H_6_N_2_O)(H_2_O)_2_], (III) (Hökelek *et al.*, 2010*e*), [Co(C_8_H_7_O_3_)_2_(C_6_H_6_N_2_O)_2_(H_2_O)_2_].2H_2_O, (IV) (Hökelek *et al.*, 2010*b*), [Ni(C_8_H_7_O_3_)_2_(C_6_H_6_N_2_O)_2_(H_2_O)_2_].2H_2_O, (V) (Hökelek *et al.*, 2010*a*), [Zn(C_8_H_8_NO_2_)_2_(C_6_H_6_N_2_O)_2_].H_2_O, (VI) (Hökelek *et al.*, 2009*a*), [Zn(C_9_H_10_NO_2_)_2_(C_6_H_6_N_2_O)_2_(H_2_O)_2_], (VII) (Hökelek *et al.*, 2009*b*) and [Zn(C_8_H_7_O_3_)_2_(C_6_H_6_N_2_O)_2_], (VIII) (Hökelek *et al.*, 2010*d*) have also been determined.

In the title compound (Fig. 1), two Cu—O bond distances [2.4581 (14) and 2.2397 (14) Å] are significantly longer than the other two, and the average Cu—O bond length is 2.1835 (14) Å. The Cu1 atom is displaced out of the least-squares planes of the carboxylate groups (O1/C1/O2), (O3/C8/O4) and (O5/C15/O6) by 0.0717 (3), 0.6121 (3) and 0.7391 (3) Å, respectively. The intramolecular O—H···O hydrogen bond links the monodentately coordinated (PFB) and (PFBA) ligands (Table 1). The dihedral angles between the planar carboxylate groups and the adjacent benzene rings A (C2–C7), B (C9–C14) and C (C16–C21) are 11.08 (14), 7.62 (13) and 5.73 (11)°, respectively, while those between rings A, B, C, D (N1/C22–C26), E (N3/C28–C32) and F (Cu1/O1/C1/O2) are A/B = 15.62 (6), A/C = 33.71 (8), A/D = 78.60 (6), A/E = 81.00 (6), A/F = 11.19 (6), B/C = 26.60 (8), B/D = 70.02 (6), B/E = 86.56 (6), B/F = 23.92 (6), C/D = 44.98 (6), C/E = 66.30 (6), C/F = 32.33 (6), D/E = 26.76 (6) and D/F = 75.15 (5)°.

In (I), the O1—Cu1—O2 angle is 57.75 (2)°. The corresponding O—M—O (where M is a metal) angles are 60.32 (4)° in (III), 59.02 (8)° in (VI), 60.03 (6)° in (VII), 57.53 (5)°, 56.19 (5)° and 59.04 (4)° in (VIII) and 55.2 (1)° in [Cu(Asp)_2_(py)_2_] (where Asp is acetylsalicylate and py is pyridine) [(IX); Greenaway *et al.*, 1984].

In the crystal structure, intermolecular N—H···O, C—H···F and C—H···O hydrogen bonds link the molecules into a three-dimensional network (Table 1 and Fig. 2). The π–π contacts between the benzene rings, Cg1–Cg1^i^, Cg1–Cg2^ii^ and Cg2–Cg3^iii^ [symmetry codes: (i) 1 - x, -y, -z, (ii) 1 + x, y, z, (iii) -x, -y, 1 - z, where Cg1, Cg2 and Cg3 are the centroids of the rings A (C2–C7), B (C9–C14) and C (C16–C21), respectively] may also stabilize the structure, with centroid–centroid distances of 3.5517 (15), 3.8456 (14) and 3.9265 (13) Å, respectively.

### Experimental

The title compound was prepared by the reaction of CuSO_4_.5H_2_O (1.23 g, 5 mmol) in H_2_O (20 ml) and NA (1.22 g, 10 mmol) in H_2_O (20 ml) with sodium 4-fluorobenzoate (1.62 g, 10 mmol) in H_2_O (50 ml) at room temperature. The mixture was filtered and set aside to crystallize at ambient temperature for one month, giving blue single crystals.

### Refinement

Atoms H2A, H2B, H4A and H4B (for NH_2_ groups) and H41 (for OH group) were located in a difference Fourier map and were freely refined. The C-bound H atoms were positioned geometrically with C—H = 0.95 Å for aromatic H atoms, and constrained to ride on their parent atoms, with *U*_iso_(H) = 1.2*U*_eq_(C).

### Crystal data

**Table:**

[Cu(C_7_H_4_FO_2_)_2_(C_7_H_5_FO_2_)(C_6_H_6_N_2_O)_2_]	*Z* = 2
*M**_r_* = 726.12	*F*(000) = 742
Triclinic, *P*1	*D*_x_ = 1.573 Mg m^−^^3^
Hall symbol: -P 1	Mo *K*α radiation, λ = 0.71073 Å
*a* = 10.3370 (2) Å	Cell parameters from 9894 reflections
*b* = 11.6707 (3) Å	θ = 2.5–28.4°
*c* = 14.1121 (4) Å	µ = 0.79 mm^−^^1^
α = 110.824 (4)°	*T* = 100 K
β = 101.333 (3)°	Block, blue
γ = 95.761 (2)°	0.48 × 0.32 × 0.24 mm
*V* = 1533.09 (8) Å^3^	

### Data collection

**Table:**

Bruker APEXII Kappa CCD area-detector diffractometer	7686 independent reflections
Radiation source: fine-focus sealed tube	6584 reflections with *I* > 2σ(*I*)
graphite	*R*_int_ = 0.031
φ and ω scans	θ_max_ = 28.5°, θ_min_ = 1.6°
Absorption correction: multi-scan (*SADABS*; Bruker, 2005)	*h* = −13→12
*T*_min_ = 0.745, *T*_max_ = 0.827	*k* = −15→15
27657 measured reflections	*l* = −18→18

### Refinement

**Table:**

Refinement on *F*^2^	Primary atom site location: structure-invariant direct methods
Least-squares matrix: full	Secondary atom site location: difference Fourier map
*R*[*F*^2^ > 2σ(*F*^2^)] = 0.036	Hydrogen site location: inferred from neighbouring sites
*wR*(*F*^2^) = 0.087	H atoms treated by a mixture of independent and constrained refinement
*S* = 1.08	*w* = 1/[σ^2^(*F*_o_^2^) + (0.0247*P*)^2^ + 1.9077*P*] where *P* = (*F*_o_^2^ + 2*F*_c_^2^)/3
7686 reflections	(Δ/σ)_max_ < 0.001
461 parameters	Δρ_max_ = 0.59 e Å^−^^3^
1 restraint	Δρ_min_ = −0.53 e Å^−^^3^

### Special details

**Table:**

Geometry. All esds (except the esd in the dihedral angle between two l.s. planes) are estimated using the full covariance matrix. The cell esds are taken into account individually in the estimation of esds in distances, angles and torsion angles; correlations between esds in cell parameters are only used when they are defined by crystal symmetry. An approximate (isotropic) treatment of cell esds is used for estimating esds involving l.s. planes.
Refinement. Refinement of F^2^ against ALL reflections. The weighted R-factor wR and goodness of fit S are based on F^2^, conventional R-factors R are based on F, with F set to zero for negative F^2^. The threshold expression of F^2^ > 2sigma(F^2^) is used only for calculating R-factors(gt) etc. and is not relevant to the choice of reflections for refinement. R-factors based on F^2^ are statistically about twice as large as those based on F, and R- factors based on ALL data will be even larger.

### Fractional atomic coordinates and isotropic or equivalent isotropic displacement parameters (Å^2^)

**Table:**

	*x*	*y*	*z*	*U*_iso_*/*U*_eq_	
Cu1	0.13629 (2)	0.00940 (2)	0.301857 (18)	0.01112 (7)	
O1	0.21519 (13)	−0.08538 (12)	0.17898 (10)	0.0128 (3)	
O2	0.37085 (14)	0.06815 (12)	0.30046 (11)	0.0147 (3)	
O3	−0.06751 (14)	−0.11248 (13)	0.23335 (11)	0.0162 (3)	
O4	−0.19206 (16)	−0.02059 (14)	0.34144 (13)	0.0243 (3)	
H41	−0.136 (3)	0.048 (2)	0.367 (3)	0.056*	
O5	0.15186 (15)	0.14357 (13)	0.43863 (11)	0.0181 (3)	
O6	−0.05429 (16)	0.19004 (14)	0.42197 (13)	0.0258 (4)	
O7	0.36816 (15)	−0.47531 (13)	0.40855 (12)	0.0205 (3)	
O8	0.08320 (16)	0.47994 (13)	0.11418 (12)	0.0240 (3)	
N1	0.19980 (16)	−0.10554 (14)	0.37023 (12)	0.0121 (3)	
N2	0.49317 (18)	−0.32379 (16)	0.55914 (14)	0.0166 (4)	
H2A	0.532 (3)	−0.377 (3)	0.572 (2)	0.036 (8)*	
H2B	0.517 (3)	−0.243 (3)	0.602 (2)	0.037 (8)*	
N3	0.09164 (16)	0.12271 (14)	0.22622 (13)	0.0121 (3)	
N4	−0.05759 (19)	0.33049 (17)	−0.02899 (14)	0.0180 (4)	
H4A	−0.094 (3)	0.260 (3)	−0.061 (2)	0.019 (6)*	
H4B	−0.070 (2)	0.380 (2)	−0.056 (2)	0.022 (6)*	
F1	0.71007 (14)	−0.12837 (14)	−0.02727 (11)	0.0314 (3)	
F2	−0.56952 (14)	−0.53850 (12)	0.11468 (12)	0.0331 (3)	
F3	0.28179 (16)	0.69704 (12)	0.76409 (12)	0.0398 (4)	
C1	0.33457 (19)	−0.02071 (17)	0.21304 (15)	0.0123 (4)	
C2	0.4321 (2)	−0.05193 (17)	0.14653 (15)	0.0137 (4)	
C3	0.4058 (2)	−0.16158 (18)	0.05765 (16)	0.0168 (4)	
H3	0.3252	−0.2172	0.0377	0.020*	
C4	0.4999 (2)	−0.1880 (2)	−0.00125 (17)	0.0218 (4)	
H4	0.4835	−0.2610	−0.0607	0.026*	
C5	0.6176 (2)	−0.1036 (2)	0.03036 (18)	0.0214 (4)	
C6	0.6464 (2)	0.0058 (2)	0.11703 (18)	0.0219 (4)	
H6	0.7266	0.0616	0.1357	0.026*	
C7	0.5526 (2)	0.03054 (18)	0.17561 (17)	0.0175 (4)	
H7	0.5705	0.1034	0.2353	0.021*	
C8	−0.1680 (2)	−0.11385 (18)	0.26792 (15)	0.0155 (4)	
C9	−0.2758 (2)	−0.22678 (18)	0.22622 (16)	0.0160 (4)	
C10	−0.2572 (2)	−0.33826 (19)	0.15506 (17)	0.0202 (4)	
H10	−0.1778	−0.3417	0.1333	0.024*	
C11	−0.3561 (2)	−0.4443 (2)	0.11633 (18)	0.0243 (5)	
H11	−0.3441	−0.5194	0.0693	0.029*	
C12	−0.4729 (2)	−0.4351 (2)	0.14966 (18)	0.0222 (4)	
C13	−0.4959 (2)	−0.3259 (2)	0.21821 (17)	0.0210 (4)	
H13	−0.5767	−0.3224	0.2377	0.025*	
C14	−0.3952 (2)	−0.22134 (19)	0.25738 (16)	0.0178 (4)	
H14	−0.4077	−0.1468	0.3050	0.021*	
C15	0.0698 (2)	0.21594 (18)	0.46296 (16)	0.0168 (4)	
C16	0.1269 (2)	0.34387 (18)	0.54508 (16)	0.0161 (4)	
C17	0.0416 (2)	0.42600 (19)	0.57831 (17)	0.0202 (4)	
H17	−0.0507	0.4006	0.5507	0.024*	
C18	0.0935 (2)	0.5457 (2)	0.65259 (18)	0.0255 (5)	
H18	0.0372	0.6013	0.6757	0.031*	
C19	0.2303 (2)	0.57979 (19)	0.69091 (17)	0.0249 (5)	
C20	0.3180 (2)	0.5021 (2)	0.65898 (17)	0.0226 (4)	
H20	0.4103	0.5289	0.6858	0.027*	
C21	0.2647 (2)	0.38235 (19)	0.58543 (16)	0.0187 (4)	
H21	0.3218	0.3274	0.5630	0.022*	
C22	0.2985 (2)	−0.06282 (17)	0.45817 (15)	0.0139 (4)	
H22	0.3234	0.0229	0.4955	0.017*	
C23	0.3654 (2)	−0.14142 (17)	0.49619 (15)	0.0147 (4)	
H23	0.4332	−0.1088	0.5579	0.018*	
C24	0.32900 (19)	−0.26950 (17)	0.44009 (15)	0.0123 (4)	
C25	0.2243 (2)	−0.31342 (17)	0.35045 (15)	0.0147 (4)	
H25	0.1963	−0.3987	0.3123	0.018*	
C26	0.1621 (2)	−0.22944 (17)	0.31836 (15)	0.0144 (4)	
H26	0.0913	−0.2599	0.2586	0.017*	
C27	0.3992 (2)	−0.36452 (17)	0.46928 (15)	0.0139 (4)	
C28	−0.00464 (19)	0.08500 (17)	0.13710 (15)	0.0127 (4)	
H28	−0.0564	0.0055	0.1121	0.015*	
C29	−0.03050 (19)	0.15950 (17)	0.08046 (15)	0.0133 (4)	
H29	−0.0996	0.1312	0.0197	0.016*	
C30	0.04892 (19)	0.27753 (17)	0.11612 (15)	0.0126 (4)	
C31	0.1513 (2)	0.31490 (17)	0.20657 (16)	0.0147 (4)	
H31	0.2075	0.3922	0.2313	0.018*	
C32	0.1690 (2)	0.23614 (17)	0.25979 (15)	0.0141 (4)	
H32	0.2369	0.2626	0.3211	0.017*	
C33	0.0257 (2)	0.37055 (17)	0.06562 (16)	0.0154 (4)	

### Atomic displacement parameters (Å^2^)

**Table:**

	*U*^11^	*U*^22^	*U*^33^	*U*^12^	*U*^13^	*U*^23^
Cu1	0.01402 (12)	0.00961 (11)	0.01052 (12)	0.00377 (8)	0.00219 (9)	0.00485 (8)
O1	0.0124 (7)	0.0113 (6)	0.0131 (7)	0.0005 (5)	0.0013 (5)	0.0043 (5)
O2	0.0155 (7)	0.0117 (6)	0.0130 (7)	0.0006 (5)	0.0008 (6)	0.0024 (5)
O3	0.0151 (7)	0.0160 (7)	0.0175 (7)	0.0018 (5)	0.0030 (6)	0.0074 (6)
O4	0.0235 (8)	0.0179 (7)	0.0240 (8)	−0.0022 (6)	0.0084 (7)	−0.0005 (6)
O5	0.0260 (8)	0.0143 (7)	0.0133 (7)	0.0089 (6)	0.0033 (6)	0.0041 (6)
O6	0.0199 (8)	0.0154 (7)	0.0327 (9)	0.0009 (6)	0.0035 (7)	0.0006 (6)
O7	0.0236 (8)	0.0110 (6)	0.0198 (8)	0.0033 (6)	−0.0060 (6)	0.0036 (6)
O8	0.0351 (9)	0.0116 (7)	0.0188 (8)	−0.0032 (6)	−0.0073 (7)	0.0078 (6)
N1	0.0147 (8)	0.0118 (7)	0.0111 (8)	0.0038 (6)	0.0036 (6)	0.0052 (6)
N2	0.0185 (9)	0.0111 (8)	0.0168 (9)	0.0033 (7)	−0.0033 (7)	0.0053 (7)
N3	0.0127 (8)	0.0110 (7)	0.0133 (8)	0.0033 (6)	0.0028 (6)	0.0053 (6)
N4	0.0256 (10)	0.0099 (8)	0.0158 (9)	−0.0006 (7)	−0.0027 (8)	0.0071 (7)
F1	0.0269 (7)	0.0399 (8)	0.0329 (8)	0.0128 (6)	0.0196 (6)	0.0125 (6)
F2	0.0289 (8)	0.0209 (7)	0.0419 (9)	−0.0082 (6)	0.0086 (7)	0.0067 (6)
F3	0.0500 (10)	0.0157 (6)	0.0340 (8)	−0.0035 (6)	−0.0018 (7)	−0.0045 (6)
C1	0.0146 (9)	0.0109 (8)	0.0121 (9)	0.0033 (7)	0.0013 (7)	0.0063 (7)
C2	0.0150 (9)	0.0139 (9)	0.0143 (9)	0.0045 (7)	0.0032 (8)	0.0076 (7)
C3	0.0164 (10)	0.0172 (9)	0.0149 (10)	0.0026 (8)	0.0021 (8)	0.0051 (8)
C4	0.0250 (11)	0.0230 (10)	0.0164 (11)	0.0088 (9)	0.0054 (9)	0.0053 (8)
C5	0.0213 (11)	0.0276 (11)	0.0229 (11)	0.0122 (9)	0.0116 (9)	0.0132 (9)
C6	0.0176 (10)	0.0219 (10)	0.0307 (12)	0.0043 (8)	0.0095 (9)	0.0132 (9)
C7	0.0175 (10)	0.0137 (9)	0.0202 (11)	0.0013 (7)	0.0048 (8)	0.0055 (8)
C8	0.0170 (10)	0.0163 (9)	0.0131 (9)	0.0023 (8)	0.0008 (8)	0.0074 (8)
C9	0.0171 (10)	0.0153 (9)	0.0154 (10)	0.0004 (7)	0.0018 (8)	0.0074 (8)
C10	0.0197 (11)	0.0185 (10)	0.0214 (11)	0.0013 (8)	0.0064 (9)	0.0064 (8)
C11	0.0276 (12)	0.0171 (10)	0.0238 (12)	0.0003 (9)	0.0068 (10)	0.0038 (9)
C12	0.0200 (11)	0.0183 (10)	0.0249 (12)	−0.0051 (8)	0.0020 (9)	0.0084 (9)
C13	0.0166 (10)	0.0248 (11)	0.0232 (11)	0.0018 (8)	0.0047 (9)	0.0118 (9)
C14	0.0203 (10)	0.0174 (9)	0.0166 (10)	0.0045 (8)	0.0044 (8)	0.0075 (8)
C15	0.0238 (11)	0.0132 (9)	0.0144 (10)	0.0035 (8)	0.0065 (8)	0.0055 (8)
C16	0.0218 (10)	0.0134 (9)	0.0149 (10)	0.0042 (8)	0.0076 (8)	0.0056 (8)
C17	0.0205 (11)	0.0178 (10)	0.0193 (11)	0.0030 (8)	0.0069 (9)	0.0027 (8)
C18	0.0311 (13)	0.0159 (10)	0.0261 (12)	0.0077 (9)	0.0092 (10)	0.0021 (9)
C19	0.0357 (13)	0.0126 (9)	0.0188 (11)	−0.0009 (9)	0.0018 (10)	0.0013 (8)
C20	0.0211 (11)	0.0219 (10)	0.0209 (11)	−0.0028 (8)	0.0004 (9)	0.0080 (9)
C21	0.0216 (11)	0.0184 (10)	0.0176 (10)	0.0050 (8)	0.0066 (9)	0.0073 (8)
C22	0.0182 (10)	0.0108 (8)	0.0113 (9)	0.0028 (7)	0.0026 (8)	0.0030 (7)
C23	0.0170 (10)	0.0126 (8)	0.0113 (9)	0.0014 (7)	−0.0013 (8)	0.0038 (7)
C24	0.0133 (9)	0.0120 (8)	0.0133 (9)	0.0034 (7)	0.0037 (8)	0.0065 (7)
C25	0.0166 (10)	0.0106 (8)	0.0162 (10)	0.0017 (7)	0.0022 (8)	0.0053 (7)
C26	0.0145 (9)	0.0130 (9)	0.0127 (9)	0.0007 (7)	−0.0010 (8)	0.0043 (7)
C27	0.0145 (9)	0.0111 (8)	0.0158 (10)	0.0027 (7)	0.0014 (8)	0.0059 (7)
C28	0.0130 (9)	0.0101 (8)	0.0141 (9)	0.0011 (7)	0.0027 (8)	0.0041 (7)
C29	0.0131 (9)	0.0135 (9)	0.0116 (9)	0.0019 (7)	−0.0002 (7)	0.0047 (7)
C30	0.0150 (9)	0.0103 (8)	0.0127 (9)	0.0032 (7)	0.0030 (8)	0.0048 (7)
C31	0.0152 (10)	0.0100 (8)	0.0173 (10)	0.0011 (7)	0.0015 (8)	0.0049 (7)
C32	0.0138 (9)	0.0125 (8)	0.0131 (9)	0.0020 (7)	−0.0013 (8)	0.0039 (7)
C33	0.0202 (10)	0.0119 (8)	0.0141 (10)	0.0019 (7)	0.0017 (8)	0.0065 (7)

### Geometric parameters (Å, °)

**Table:**

Cu1—O1	2.0661 (13)	C9—C10	1.391 (3)
Cu1—O2	2.4581 (14)	C9—C14	1.389 (3)
Cu1—O3	2.2397 (14)	C10—C11	1.386 (3)
Cu1—O5	1.9701 (14)	C10—H10	0.9300
Cu1—N1	2.0024 (15)	C11—C12	1.380 (3)
Cu1—N3	2.0084 (15)	C11—H11	0.9300
O1—C1	1.283 (2)	C13—C12	1.375 (3)
O2—C1	1.252 (2)	C13—C14	1.384 (3)
O3—C8	1.233 (2)	C13—H13	0.9300
O4—C8	1.294 (2)	C14—H14	0.9300
O4—H41	0.855 (18)	C16—C15	1.498 (3)
O5—C15	1.266 (2)	C16—C17	1.389 (3)
O6—C15	1.256 (3)	C16—C21	1.387 (3)
O7—C27	1.237 (2)	C17—H17	0.9300
O8—C33	1.232 (2)	C18—C17	1.388 (3)
N1—C22	1.338 (3)	C18—H18	0.9300
N1—C26	1.342 (2)	C19—C18	1.372 (3)
N2—C27	1.331 (3)	C20—C19	1.372 (3)
N2—H2A	0.83 (3)	C20—C21	1.387 (3)
N2—H2B	0.89 (3)	C20—H20	0.9300
N3—C28	1.339 (2)	C21—H21	0.9300
N3—C32	1.345 (2)	C22—H22	0.9300
N4—C33	1.329 (3)	C23—C22	1.393 (3)
N4—H4A	0.79 (3)	C23—C24	1.390 (3)
N4—H4B	0.81 (3)	C23—H23	0.9300
F1—C5	1.361 (2)	C24—C27	1.515 (2)
F2—C12	1.356 (2)	C25—C24	1.387 (3)
F3—C19	1.362 (2)	C25—C26	1.381 (3)
C1—C2	1.494 (3)	C25—H25	0.9300
C2—C3	1.393 (3)	C26—H26	0.9300
C2—C7	1.388 (3)	C28—C29	1.387 (2)
C3—H3	0.9300	C28—H28	0.9300
C4—C3	1.388 (3)	C29—H29	0.9300
C4—C5	1.372 (3)	C30—C31	1.385 (3)
C4—H4	0.9300	C30—C29	1.394 (3)
C6—C5	1.373 (3)	C31—C32	1.383 (3)
C6—C7	1.381 (3)	C31—H31	0.9300
C6—H6	0.9300	C32—H32	0.9300
C7—H7	0.9300	C33—C30	1.510 (2)
C9—C8	1.490 (3)		
			
O1—Cu1—O3	95.03 (5)	C12—C13—C14	118.1 (2)
O1—Cu1—O2	57.75 (5)	C12—C13—H13	120.9
O5—Cu1—O1	151.82 (6)	C14—C13—H13	120.9
O5—Cu1—O3	113.15 (6)	C9—C14—H14	119.7
O5—Cu1—N1	91.60 (6)	C13—C14—C9	120.56 (19)
O5—Cu1—N3	91.70 (6)	C13—C14—H14	119.7
N1—Cu1—O1	87.92 (6)	O5—C15—C16	116.89 (19)
N1—Cu1—O3	90.60 (6)	O6—C15—O5	125.26 (19)
N1—Cu1—N3	173.23 (7)	O6—C15—C16	117.83 (18)
N3—Cu1—O1	86.39 (6)	C17—C16—C15	119.70 (19)
N3—Cu1—O3	93.54 (6)	C21—C16—C15	120.46 (18)
C1—O1—Cu1	98.92 (11)	C21—C16—C17	119.79 (19)
C8—O3—Cu1	131.63 (13)	C16—C17—H17	119.9
C8—O4—H41	118 (2)	C18—C17—C16	120.3 (2)
C15—O5—Cu1	127.21 (13)	C18—C17—H17	119.9
C22—N1—Cu1	120.76 (13)	C17—C18—H18	121.0
C22—N1—C26	118.27 (16)	C19—C18—C17	118.1 (2)
C26—N1—Cu1	119.79 (13)	C19—C18—H18	121.0
C27—N2—H2A	116 (2)	F3—C19—C18	118.3 (2)
C27—N2—H2B	123.0 (18)	F3—C19—C20	118.2 (2)
H2A—N2—H2B	121 (3)	C18—C19—C20	123.4 (2)
C28—N3—Cu1	122.20 (12)	C19—C20—C21	117.9 (2)
C28—N3—C32	118.18 (16)	C19—C20—H20	121.1
C32—N3—Cu1	119.29 (13)	C21—C20—H20	121.1
C33—N4—H4A	123.1 (18)	C16—C21—H21	119.7
C33—N4—H4B	118.4 (18)	C20—C21—C16	120.6 (2)
H4A—N4—H4B	118 (3)	C20—C21—H21	119.7
O1—C1—C2	119.12 (17)	N1—C22—C23	122.71 (17)
O2—C1—O1	121.48 (18)	N1—C22—H22	118.6
O2—C1—C2	119.40 (17)	C23—C22—H22	118.6
C3—C2—C1	121.88 (18)	C22—C23—H23	120.7
C7—C2—C1	118.57 (18)	C24—C23—C22	118.69 (18)
C7—C2—C3	119.54 (19)	C24—C23—H23	120.7
C2—C3—H3	119.9	C23—C24—C27	123.94 (17)
C4—C3—C2	120.1 (2)	C25—C24—C23	118.34 (17)
C4—C3—H3	119.9	C25—C24—C27	117.70 (17)
C3—C4—H4	120.8	C24—C25—H25	120.3
C5—C4—C3	118.3 (2)	C26—C25—C24	119.47 (18)
C5—C4—H4	120.8	C26—C25—H25	120.3
F1—C5—C4	118.8 (2)	N1—C26—C25	122.45 (18)
F1—C5—C6	118.1 (2)	N1—C26—H26	118.8
C4—C5—C6	123.2 (2)	C25—C26—H26	118.8
C5—C6—C7	118.0 (2)	O7—C27—N2	123.15 (17)
C5—C6—H6	121.0	O7—C27—C24	118.91 (17)
C7—C6—H6	121.0	N2—C27—C24	117.93 (17)
C2—C7—H7	119.6	N3—C28—C29	122.77 (17)
C6—C7—C2	120.9 (2)	N3—C28—H28	118.6
C6—C7—H7	119.6	C29—C28—H28	118.6
O3—C8—O4	124.89 (19)	C28—C29—C30	118.84 (18)
O3—C8—C9	121.66 (18)	C28—C29—H29	120.6
O4—C8—C9	113.45 (18)	C30—C29—H29	120.6
C10—C9—C8	119.82 (19)	C29—C30—C33	124.01 (17)
C14—C9—C8	120.45 (18)	C31—C30—C29	118.32 (17)
C14—C9—C10	119.73 (19)	C31—C30—C33	117.58 (17)
C9—C10—H10	119.8	C30—C31—H31	120.3
C11—C10—C9	120.4 (2)	C32—C31—C30	119.37 (18)
C11—C10—H10	119.8	C32—C31—H31	120.3
C10—C11—H11	121.0	N3—C32—C31	122.47 (18)
C12—C11—C10	118.0 (2)	N3—C32—H32	118.8
C12—C11—H11	121.0	C31—C32—H32	118.8
F2—C12—C11	118.9 (2)	O8—C33—N4	123.07 (18)
F2—C12—C13	118.0 (2)	O8—C33—C30	118.78 (17)
C13—C12—C11	123.1 (2)	N4—C33—C30	118.15 (17)
			
O3—Cu1—O1—C1	−179.56 (10)	C7—C6—C5—C4	1.0 (3)
O5—Cu1—O1—C1	0.34 (17)	C5—C6—C7—C2	−1.1 (3)
N1—Cu1—O1—C1	−89.15 (11)	C10—C9—C8—O3	7.4 (3)
N3—Cu1—O1—C1	87.19 (11)	C10—C9—C8—O4	−173.04 (18)
O1—Cu1—O3—C8	176.08 (16)	C14—C9—C8—O3	−172.14 (18)
O5—Cu1—O3—C8	−3.87 (18)	C14—C9—C8—O4	7.4 (3)
N1—Cu1—O3—C8	88.12 (17)	C8—C9—C10—C11	179.4 (2)
N3—Cu1—O3—C8	−97.24 (17)	C14—C9—C10—C11	−1.0 (3)
O1—Cu1—O5—C15	133.63 (16)	C8—C9—C14—C13	179.55 (19)
O3—Cu1—O5—C15	−46.47 (17)	C10—C9—C14—C13	0.0 (3)
N1—Cu1—O5—C15	−137.80 (17)	C9—C10—C11—C12	0.7 (3)
N3—Cu1—O5—C15	48.11 (17)	C10—C11—C12—F2	−178.4 (2)
O1—Cu1—N1—C22	112.45 (15)	C10—C11—C12—C13	0.7 (3)
O1—Cu1—N1—C26	−54.95 (15)	C14—C13—C12—F2	177.43 (19)
O3—Cu1—N1—C22	−152.53 (15)	C14—C13—C12—C11	−1.7 (3)
O3—Cu1—N1—C26	40.06 (15)	C12—C13—C14—C9	1.3 (3)
O5—Cu1—N1—C22	−39.36 (15)	C17—C16—C15—O5	−177.41 (19)
O5—Cu1—N1—C26	153.23 (15)	C17—C16—C15—O6	4.4 (3)
O1—Cu1—N3—C28	70.36 (15)	C21—C16—C15—O5	4.9 (3)
O1—Cu1—N3—C32	−102.90 (15)	C21—C16—C15—O6	−173.34 (19)
O3—Cu1—N3—C28	−24.46 (15)	C15—C16—C17—C18	−178.52 (19)
O3—Cu1—N3—C32	162.27 (14)	C21—C16—C17—C18	−0.8 (3)
O5—Cu1—N3—C28	−137.78 (15)	C15—C16—C21—C20	177.92 (19)
O5—Cu1—N3—C32	48.95 (15)	C17—C16—C21—C20	0.2 (3)
Cu1—O1—C1—O2	2.02 (18)	C19—C18—C17—C16	0.4 (3)
Cu1—O1—C1—C2	−177.69 (13)	F3—C19—C18—C17	−179.8 (2)
Cu1—O3—C8—O4	21.5 (3)	C20—C19—C18—C17	0.6 (4)
Cu1—O3—C8—C9	−159.07 (13)	C21—C20—C19—F3	179.21 (19)
Cu1—O5—C15—O6	28.1 (3)	C21—C20—C19—C18	−1.1 (3)
Cu1—O5—C15—C16	−149.96 (14)	C19—C20—C21—C16	0.7 (3)
Cu1—N1—C22—C23	−165.88 (15)	C24—C23—C22—N1	0.5 (3)
C26—N1—C22—C23	1.7 (3)	C22—C23—C24—C25	−2.1 (3)
Cu1—N1—C26—C25	165.40 (15)	C22—C23—C24—C27	176.20 (18)
C22—N1—C26—C25	−2.3 (3)	C23—C24—C27—O7	−172.7 (2)
Cu1—N3—C28—C29	−175.61 (14)	C23—C24—C27—N2	6.4 (3)
C32—N3—C28—C29	−2.3 (3)	C25—C24—C27—O7	5.6 (3)
Cu1—N3—C32—C31	174.44 (15)	C25—C24—C27—N2	−175.24 (18)
C28—N3—C32—C31	0.9 (3)	C26—C25—C24—C23	1.6 (3)
O1—C1—C2—C3	−11.5 (3)	C26—C25—C24—C27	−176.86 (18)
O1—C1—C2—C7	169.33 (17)	C24—C25—C26—N1	0.7 (3)
O2—C1—C2—C3	168.76 (17)	N3—C28—C29—C30	1.6 (3)
O2—C1—C2—C7	−10.4 (3)	C31—C30—C29—C28	0.5 (3)
C1—C2—C3—C4	−179.11 (18)	C33—C30—C29—C28	−175.99 (18)
C7—C2—C3—C4	0.0 (3)	C29—C30—C31—C32	−1.8 (3)
C1—C2—C7—C6	179.76 (18)	C33—C30—C31—C32	174.95 (18)
C3—C2—C7—C6	0.6 (3)	C30—C31—C32—N3	1.1 (3)
C5—C4—C3—C2	−0.1 (3)	O8—C33—C30—C29	165.5 (2)
C3—C4—C5—F1	−179.61 (18)	O8—C33—C30—C31	−10.9 (3)
C3—C4—C5—C6	−0.4 (3)	N4—C33—C30—C29	−13.6 (3)
C7—C6—C5—F1	−179.78 (18)	N4—C33—C30—C31	169.95 (19)

### Hydrogen-bond geometry (Å, °)

**Table:**

*D*—H···*A*	*D*—H	H···*A*	*D*···*A*	*D*—H···*A*
N2—H2A···O7^i^	0.83 (3)	2.17 (4)	2.990 (3)	175 (3)
N2—H2B···O2^ii^	0.90 (3)	2.07 (3)	2.943 (2)	163 (3)
N4—H4A···O1^iii^	0.79 (3)	2.18 (3)	2.953 (2)	165 (3)
N4—H4B···O8^iv^	0.80 (3)	2.08 (3)	2.880 (3)	174 (2)
O4—H41···O6	0.85 (3)	1.62 (3)	2.457 (2)	169 (3)
C4—H4···F2^v^	0.93	2.50	3.248 (3)	137
C23—H23···O2^ii^	0.93	2.42	3.333 (2)	167
C25—H25···O8^vi^	0.93	2.60	3.275 (2)	130
C31—H31···O7^vii^	0.93	2.55	3.251 (3)	132

Symmetry codes: (i) −*x*+1, −*y*−1, −*z*+1; (ii) −*x*+1, −*y*, −*z*+1; (iii) −*x*, −*y*, −*z*; (iv) −*x*, −*y*+1, −*z*; (v) −*x*, −*y*−1, −*z*; (vi) *x*, *y*−1, *z*; (vii) *x*, *y*+1, *z*.
